# Supplementary material for: The effect of software and hardware version on Apple Watch activity measurement: A secondary analysis of the COVFIT retrospective cohort study
Source: PLOS Digit Health. 2025 Apr 8;4(4):e0000727. doi: 10.1371/journal.pdig.0000727 (PMC11977988; doi:10.1371/journal.pdig.0000727)
Supplement: S1 Table — (DOCX) [file pdig.0000727.s001.docx]

| **Supplementary Table 1.** Participant characteristics, overall and for each transition, among the full analytic sample (activity data for at least one day in both the pre- and post-transition week) | | | | | | | |
| --- | --- | --- | --- | --- | --- | --- | --- |
|  | Overall  n = 341 | Software transitions | | | | | Change in hardware  n = 147* |
|  |  | 5 to 6  n = 165* | 6 to 7  n = 208* | 7 to 8  n = 277* | | 8 to 9  n = 242* |  |
| **Sex = Male (%)** | 186 (54.5) | 101 (61.2) | 128 (61.5) | 157 (56.7) | 147 (60.7) | | 116 (78.9) |
| **Age (%)** | | | | | | | |
| 18-29 | 53 (15.5) | 25 (15.2) | 31 (14.9) | 34 (12.3) | 36 (14.9) | | 18 (12.2) |
| 30-49 | 165 (48.4) | 82 (49.7) | 101 (48.6) | 137 (49.5) | 116 (47.9) | | 84 (57.1) |
| 50-69 | 111 (32.6) | 55 (33.3) | 70 (33.7) | 96 (34.7) | 80 (33.1) | | 42 (28.6) |
| 70+ | 12 (3.5) | 3 (1.8) | 6 (2.9) | 10 (3.6) | 10 (4.1) | | 3 (2.0) |
| **Household income, 2019 (%)** | | | | | | | |
| Under $50,000 | 28 (8.2) | 13 (7.9) | 11 (5.3) | 16 (5.8) | 18 (7.4) | | 16 (10.9) |
| $50,000 to $99,999 | 60 (17.6) | 26 (15.8) | 37 (17.8) | 48 (17.3) | 47 (19.4) | | 20 (13.6) |
| $100,000 to $149,999 | 85 (24.9) | 39 (23.6) | 49 (23.6) | 68 (24.5) | 59 (24.4) | | 40 (27.2) |
| $150,000 to $199,999 | 62 (18.2) | 24 (14.5) | 34 (16.3) | 55 (19.9) | 45 (18.6) | | 23 (15.6) |
| $200,000 or more | 93 (27.3) | 57 (34.5) | 68 (32.7) | 81 (29.2) | 63 (26.0) | | 43 (29.3) |
| No income | 2 (0.6) | 0 (0.0) | 1 (0.5) | 2 (0.7) | 2 (0.8) | | 0 (0.0) |
| Prefer not to answer | 11 (3.2) | 6 (3.6) | 8 (3.8) | 7 (2.5) | 8 (3.3) | | 5 (3.4) |
| **Education (%)** | | | | | | | |
| Graduate degree | 124 (36.4) | 60 (36.4) | 75 (36.1) | 103 (37.2) | 87 (36.0) | | 39 (26.5) |
| Undergraduate degree | 147 (43.1) | 72 (43.6) | 94 (45.2) | 119 (43.0) | 106 (43.8) | | 73 (49.7) |
| Trade or technical school | 31 (9.1) | 14 (8.5) | 18 (8.7) | 25 (9.0) | 20 (8.3) | | 12 (8.2) |
| High school or less | 34 (10.0) | 17 (10.3) | 18 (8.7) | 27 (9.7) | 24 (9.9) | | 22 (15.0) |
| Prefer not to answer | 5 (1.5) | 2 (1.2) | 3 (1.4) | 3 (1.1) | 5 (2.1) | | 1 (0.7) |
| **Baseline daily physical activity (week before the transition) (mean (SD))** | | | | | | | |
| Exercise minutes, overall | 43.90 (34.74) | 38.28 (32.01) | 41.90 (31.42) | 39.41 (30.84) | 51.34 (40.90) | | 49.22 (35.65) |
| Exercise minutes, female | 39.69 (27.91) | 34.97 (27.56) | 37.65 (24.38) | 35.52 (26.08) | 47.84 (31.08) | | 45.86 (28.97) |
| Exercise minutes, male | 46.42 (38.06) | 40.37 (34.50) | 44.55 (34.94) | 42.40 (33.81) | 53.60 (46.12) | | 50.12 (37.29) |
| Active calories, overall | 663.91 (253.80) | 666.23 (244.45) | 670.05 (251.87) | 637.39 (244.56) | 651.62 (271.11) | | 722.82 (247.68) |
| Active calories, female | 553.89 (187.94) | 576.75 (202.91) | 564.85 (188.61) | 541.39 (182.28) | 534.02 (181.64) | | 587.71 (195.47) |
| Active calories, male | 730.02 (265.16) | 722.93 (252.37) | 735.80 (264.47) | 710.77 (260.70) | 727.62 (292.01) | | 758.93 (248.35) |
| * Data is at the transition-level, not the person-level. | | | | | | | |
