# Supplementary material for: The effect of software and hardware version on Apple Watch activity measurement: A secondary analysis of the COVFIT retrospective cohort study
Source: PLOS Digit Health. 2025 Apr 8;4(4):e0000727. doi: 10.1371/journal.pdig.0000727 (PMC11977988; doi:10.1371/journal.pdig.0000727)
Supplement: S3 Table — (DOCX) [file pdig.0000727.s003.docx]

| **Supplementary Table 3.** Change in physical activity (a) during the transition period (week before vs. week after each transition), and (b) during the control transition period (two weeks before vs. one week before each transition), overall and stratified by gender, among the full sample (activity data for at least one day in both the pre- and post- week) | | | | | |
| --- | --- | --- | --- | --- | --- |
|  | **5 to 6** | **6 to 7** | **7 to 8** | **8 to 9** | **Hardware** |
| ***Female participants*** | | | | | |
| **(a) Change in daily physical activity during the transition period (week before vs. week after transition)** | | | | | |
| Number of participants | 64 | 80 | 120 | 95 | 31 |
| Exercise minutes, mean (SD) | 0.42 (16.86) | -1.56 (19.93) | 6.02 (18.55) | 1.37 (19.79) | -5.04 (23.57) |
| Active calories, mean (SD) | -7.52 (113.25) | -11.39 (128.09) | 5.62 (108.36) | -2.38 (97.45) | -34.37 (150.26) |
| **(b) Change in mean daily physical activity during the pseudo-transition period (two weeks before vs. week before transition)** | | | | | |
| Number of participants | 60 | 78 | 117 | 95 | 28 |
| Exercise minutes, mean (SD) | 0.63 (13.33) | 2.25 (16.96) | 0.95 (15.34) | -1.11 (18.40) | 0.03 (21.02) |
| Active calories, mean (SD) | 14.76 (111.71) | 12.14 (119.03) | -3.68 (106.09) | -13.53 (96.48) | 3.61 (108.29) |
| ***Male participants*** | | | | | |
| **(a) Change in daily physical activity during the transition period (week before vs. week after transition)** | | | | | |
| Number of participants | 101 | 128 | 157 | 147 | 116 |
| Exercise minutes, mean (SD) | 2.39 (19.82) | -1.05 (15.47) | 3.77 (14.91) | -4.05 (22.78) | -0.86 (18.40) |
| Active calories, mean (SD) | 11.11 (135.74) | -14.00 (126.36) | -5.64 (122.44) | -31.69 (163.45) | -12.78 (142.53) |
| **(b) Change in mean daily physical activity during the pseudo-transition period (two weeks before vs. week before transition)** | | | | | |
| Number of participants | 99 | 123 | 155 | 144 | 102 |
| Exercise minutes, mean (SD) | -0.12 (17.70) | 1.27 (18.72) | -3.10 (15.98) | 2.20 (25.33) | 2.19 (17.18) |
| Active calories, mean (SD) | 2.71 (133.80) | 6.30 (135.87) | -15.59 (130.11) | 8.01 (172.18) | 29.58 (143.25) |
