# Supplementary material for: The effect of software and hardware version on Apple Watch activity measurement: A secondary analysis of the COVFIT retrospective cohort study
Source: PLOS Digit Health. 2025 Apr 8;4(4):e0000727. doi: 10.1371/journal.pdig.0000727 (PMC11977988; doi:10.1371/journal.pdig.0000727)
Supplement: S5 Table — (DOCX) [file pdig.0000727.s005.docx]

| **Supplementary Table 5.** Effect of each software transition on daily active calories (negative binomial models with a random intercept per participant) among participants with activity data for at least one day in the 7 days before and 7 days after the specified software transition | | | | | | |
| --- | --- | --- | --- | --- | --- | --- |
| Transition | **Overall** | | **Female participants** | | **Male participants** | |
|  | **Unadjusted RR (95% CI)** | **Adjusted RR (95% CI)** | **Unadjusted RR (95% CI)** | **Adjusted RR (95% CI)** | **Unadjusted RR (95% CI)** | **Adjusted RR (95% CI)** |
| **5 to 6** | 1.00 (0.98, 1.03) | 1.00 (0.98, 1.03) | 0.99 (0.96, 1.03) | 0.69 (0.95, 1.03) | 1.01 (0.98, 1.04) | 1.01 (0.98, 1.04) |
| **6 to 7** | 0.98 (0.96, 1.01) | 0.98 (0.96, 1.01) | 0.98 (0.95, 1.02) | 0.98 (0.94, 1.02) | 0.98 (0.96, 1.01) | 0.98 (0.95, 1.01) |
| **7 to 8** | 1.00 (0.98, 1.02) | 1.00 (0.98, 1.02) | 1.00 (0.98, 1.03) | 1.00 (0.97, 1.03) | 1.00 (0.97, 1.02) | 1.00 (0.97, 1.02) |
| **8 to 9** | 0.97 (0.95, 0.99) | 0.97 (0.95, 0.99) | 0.99 (0.96, 1.02) | 0.99 (0.96, 1.02) | 0.96 (0.93, 0.98) | 0.96 (0.93, 0.98) |
