# Supplementary material for: The effect of software and hardware version on Apple Watch activity measurement: A secondary analysis of the COVFIT retrospective cohort study
Source: PLOS Digit Health. 2025 Apr 8;4(4):e0000727. doi: 10.1371/journal.pdig.0000727 (PMC11977988; doi:10.1371/journal.pdig.0000727)
Supplement: S6 Table — (DOCX) [file pdig.0000727.s006.docx]

| **Supplementary Table 6.** Effect of hardware transitions on daily exercise minutes and daily active calories (negative binomial models with a random intercept per participant) among participants with at least one day of activity data in the 7 days before and 7 days after a hardware transition | | | | | | |
| --- | --- | --- | --- | --- | --- | --- |
|  | **Overall** | | **Female participants** | | **Male participants** | |
|  | **Unadjusted RR (95% CI)** | **Adjusted RR (95% CI)** | **Unadjusted RR (95% CI)** | **Adjusted RR (95% CI)** | **Unadjusted RR (95% CI)** | **Adjusted RR (95% CI)** |
| **Exercise minutes** | 0.94 (0.88, 1.01) | 0.94 (0.88, 1.02) | 0.94 (0.81, 1.11) | 0.94 (0.79, 1.12) | 0.94 (0.87, 1.02) | 0.94 (0.88, 1.01) |
| **Active calories** | 0.97 (0.94, 0.99) | 0.97 (0.94, 1.00) | 0.96 (0.90, 1.01) | 0.96 (0.90, 1.01) | 0.97 (0.94, 1.00) | 0.97 (0.94, 1.00) |
