# Supplementary figures and images for: The effect of software and hardware version on Apple Watch activity measurement: A secondary analysis of the COVFIT retrospective cohort study
Source: PLOS Digit Health. 2025 Apr 8;4(4):e0000727. doi: 10.1371/journal.pdig.0000727 (PMC11977988; doi:10.1371/journal.pdig.0000727)

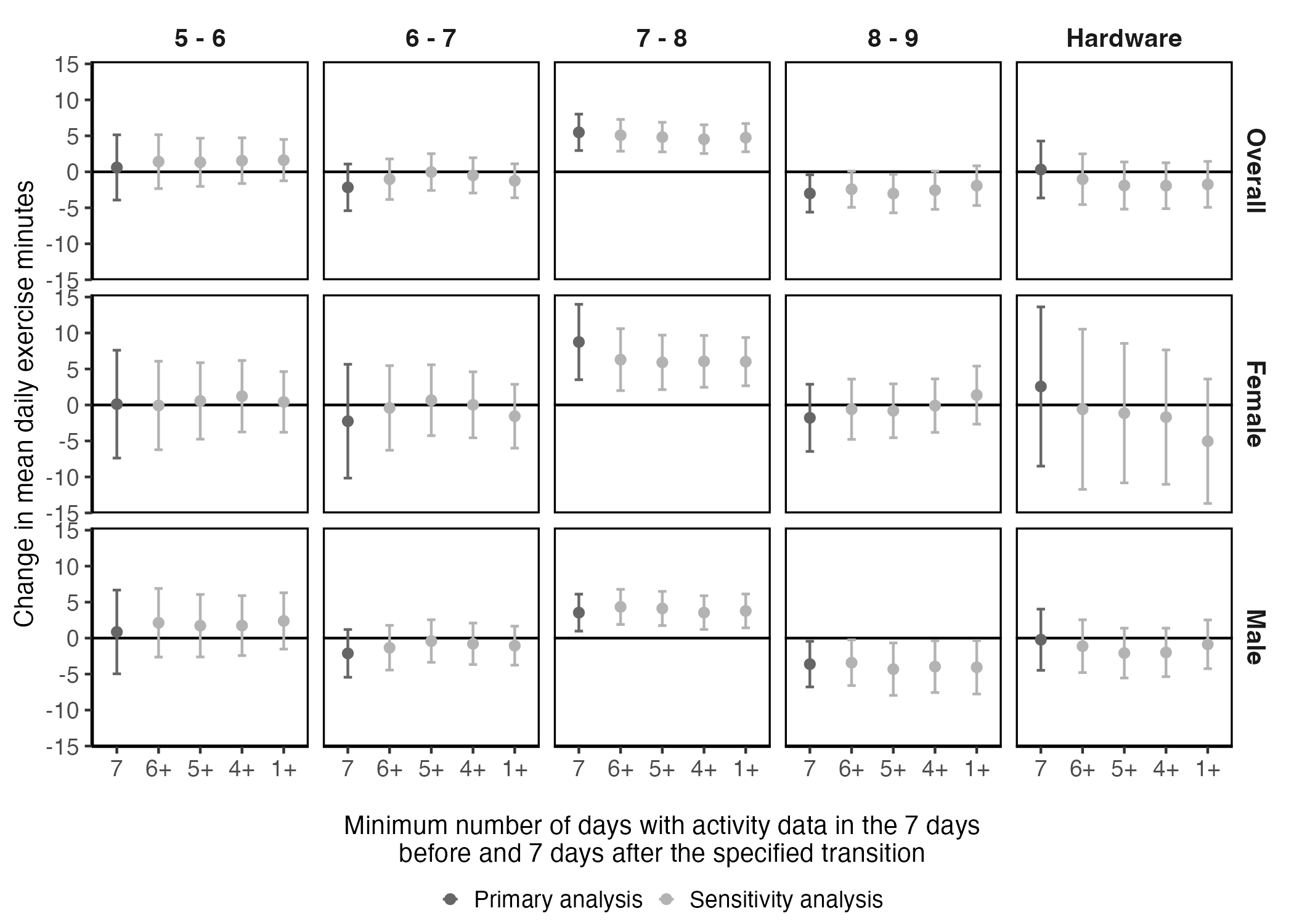

Supplement: S1 Fig — (TIFF) [file pdig.0000727.s007.tiff]

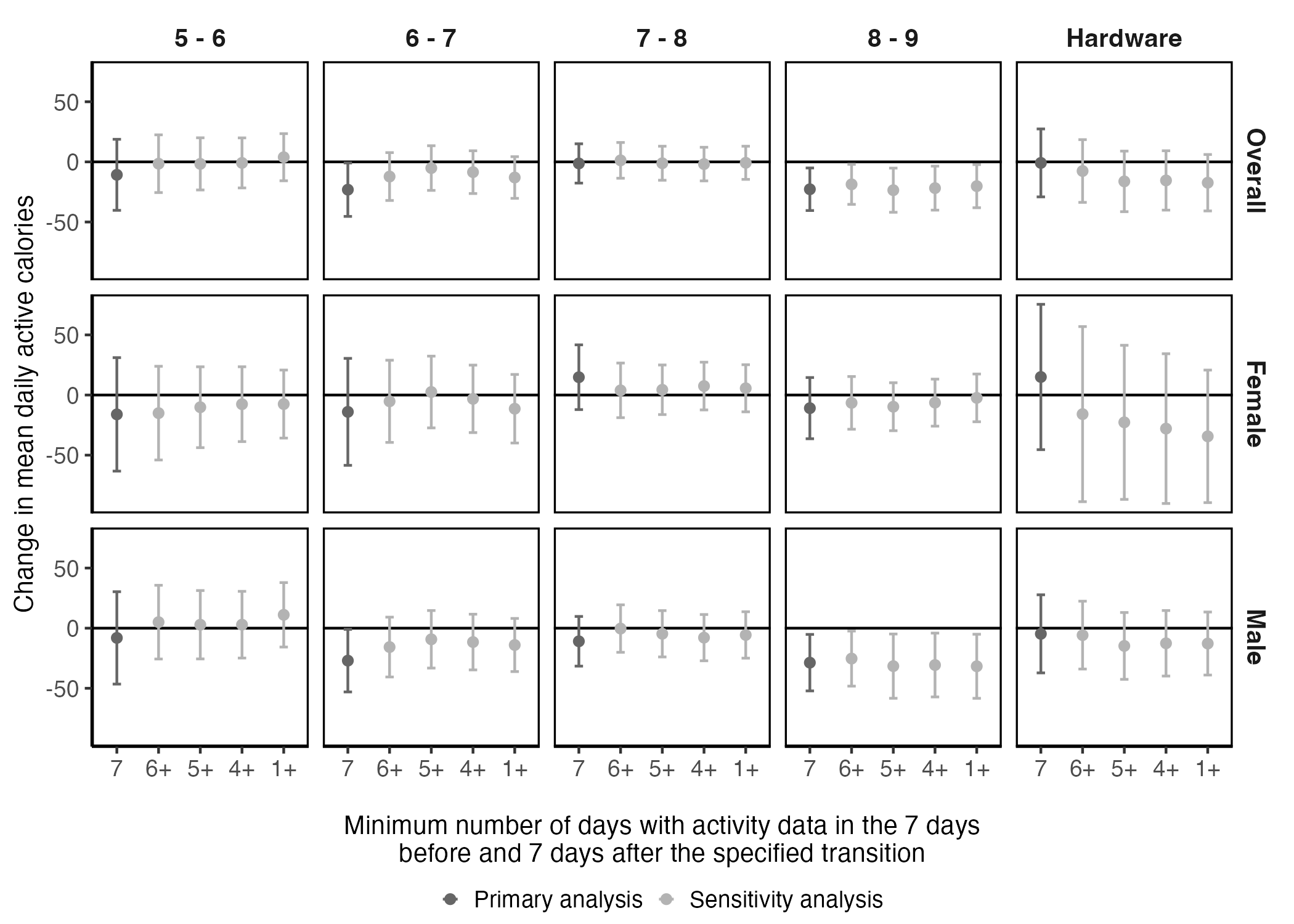

Supplement: S2 Fig — (TIFF) [file pdig.0000727.s008.tiff]
