# Supplementary material for: The effect of software and hardware version on Apple Watch activity measurement: A secondary analysis of the COVFIT retrospective cohort study
Source: PLOS Digit Health. 2025 Apr 8;4(4):e0000727. doi: 10.1371/journal.pdig.0000727 (PMC11977988; doi:10.1371/journal.pdig.0000727)
Supplement: Attachment — Submitted filename: response-to-reviewers.docx [file pdig.0000727.s010.docx]

Additional Editor Comments (if provided):

| **Editor Comment** | **Author Response** |
| --- | --- |
| The authors need to clearly state how the results could impact research, even if it seems obvious. If the research is conducted while the watch undergoes software or hardware updates, could this affect the results? | Thank you for this feedback.  We have updated the third paragraph of the introduction to make this clearer. (“However, hardware and software upgrades may also affect physical activity measurement, posing a significant threat to the validity of research leveraging consumer wearable devices. New hardware versions may contain new and/or improved sensors, while software upgrades could alter activity processing algorithms. This means that the accuracy of physical activity data from wearable devices may vary between people and/or over time depending on software or hardware version. Furthermore, comparisons of physical activity levels over time or between individuals may reflect the combined effects of differences in behaviour and differences in measurement, due to differences in software or hardware versions.”)  We also updated the first sentence of the fourth paragraph of the introduction: “While researchers have noted the potential impact of software or hardware version on activity measurement – and thus on research using commercial wearable devices^4^ – no studies have attempted to quantify this effect.” We have referenced the following paper, which clearly identifies hardware and software upgrades as a threat to the validity of research using wearable devices to measure physical activity:  Strain T, Wijndaele K, Pearce M, Brage S. Considerations for the use of consumer-grade wearables and smartphones in population surveillance of physical activity. J Meas Phys Behav. 2022;5(1):8–14.  We also revisit it in the “Research Implications” section of the discussion (“These results have important implications for physical activity research using consumer wearable devices. Most notably, failing to account for software version may introduce bias. Studies comparing physical activity levels between participants or over time, including analyses of longitudinal trends and/or the effect of an intervention, may be confounded by differences in software versions between people and/or over the course of the study period. A large majority of Apple Watch users in our sample installed all major software versions released during their study period. These upgrades are annual, so many software changes should be expected over the course of a multi-year longitudinal study. Based on our estimates, the confounding may be large and either over or underestimate the association of interest. As such, it is important that researchers using data from consumer wearables collect data about software and hardware versions when either varies between participants or over time.”).  Please let us know if this needs to be made clearer. |
| Exercise minutes and active calories are presented as percentages of change, but is it possible to show the mean and standard deviation (SD) of minutes and calories as well? This would help determine how active the cohort is. Additionally, is there a way to present the significance (p-value) of these changes? | We have added the mean and standard deviation of daily exercise minutes and mean daily active calories in the week before each of the transitions (“baseline daily exercise minutes” and “baseline daily active calories”), overall and stratified by sex, to Table 1.  We have opted to provide confidence intervals instead of p-values to illustrate the statistical significance of changes in daily exercise minutes and active calories. |
| Were all the data collected in September, when the software version was updated? | No, the data were not all from September. The two-week window was specific to each participant and was anchored around the date when that individual upgraded their software or hardware.  We have made the following changes to clarify this:  (1) We have added the following sentence to the second paragraph in the “Study design and data collection” section of the methods: **“The current study only considered physical activity data collected immediately before and immediately after the participant upgraded their hardware or software, the timing of which differed between people.”**  (2) We have revised the second paragraph of the “Data processing” paragraph of the Methods to read: “We estimated the effect of each transition of interest (i.e., from watchOS 5 to 6, 6 to 7, 7 to 8, 8 to 9 and between any two hardware versions) by comparing daily active calories and exercise minutes in the 7 days before and 7 days after (inclusive of) the upgrade. This 14-day “transition period” was unique to each participant and was centered around their transition date, defined as the first day when most of their heart rate measurements were recorded on the new software or hardware version (Figure 1, Panel A).”  (3) We also created a figure (Figure 1) to outline this process and to help visualize the statistical analysis. |
| Did the authors record data from two consecutive weeks with the same software and hardware versions? This would help clarify whether the observed changes are due to the software or hardware update, rather than normal week-to-week variations. | Thank you for this suggestion. We have added a control analysis to address this. In short, the control analysis compares physical activity two weeks before vs. one week before the transition (while the actual analysis compares physical activity in the week before and the week after the transition).  The control analysis is discussed in the following sections:  Methods:  (a) “Data processing” section:  “To estimate normal week-to-week variability, we also conducted a control analysis for each transition, comparing activity data from 15-8 days before and 7-1 day before that upgrade (“control transition period”) (Figure 1).”  (b) “Descriptive analyses”: “As a control, this was repeated comparing data from 15-8 days before and 7-1 days before each given transition, where no software or hardware upgrades occurred.”  (c) “Regression analyses”: “This analysis was conducted separately for each outcome (daily exercise minutes and daily active calories) and was repeated using data from the pseudo-transition period as a control.”  Results  For each set of results (e.g., the effect of transitioning from watchOS 7 to 8), we follow up with the results of the control analysis for that transition (two weeks before vs. one week before the watchOS 7 to 8 transition). For example, from the first paragraph of the “Software version” section of the results:  “In descriptive analyses, mean daily exercise minutes were, on average, 5.5 minutes higher in the week after vs. the week before upgrading from watchOS 7 to 8 (Supplementary Table 2; Figure 2 Panel A). The average change was larger for female (8.7 minutes) than male participants (3.5 minutes). In mixed effects negative binomial regression models, the watchOS 7 to 8 transition was associated with a 13% increase in daily exercise minutes (unadjusted rate ratio (RR) = 1.13, 95% CI: 1.06, 1.20; adjusted RR = 1.13, 95% CI: 1.06, 1.19). The effect was similar in sex-stratified models (Table 3). **Importantly, there was no significant change in daily exercise minutes in the control analysis comparing data from two weeks before vs. one week before the watchOS 5 to 6 upgrade (Figure 2 Panel B; Table 3).**”  We have also updated Supplementary Table 2 (previously part of Table 1) to include the change in mean daily exercise minutes and mean daily active calories across this control transition period (2 weeks before vs. one week before) for each transition of interest, and we have updated Figures 2 and 3 (previously Figures 1 and 2) to include the effect of each transition on daily exercise minutes, as well as the associated change in daily exercise minutes in the two weeks before that transition.  We have also added a paragraph to the discussion, highlighting how the null results of the control analysis (for most transitions) strengthens our conclusion that the software transitions may be altering physical activity measurement. |
| Are the differences between men and women influenced by variations in their weights and body sizes? | We have added the following sentence to the third paragraph of the discussion: “Third, the observed changes may be due to updates in how activity algorithms account for weight, which differs on average by sex.” |
| Was sleep time included in the total time the watch was worn? | Yes, any period when the user’s Apple Watch was worn (heart rate measurements less than 40 minutes apart) would be included in the total daily wear time. |
| The statistical analysis needs to be explained more clearly. | We have added sub-headings to the “Statistical analysis” section to differentiate between the “Descriptive analyses” and “Regression analyses”. |
| It is recommended to separate Table 1 into two tables, with one focusing on participant characteristics | We have split Table 1 into Table 1 and Supplementary Table 2, where the former contains participant characteristics and the latter summarizes changes in the two outcomes observed when comparing the week before and after the upgrade of interest. |

Review Comments to the Author.

**Reviewer #1:**

| **Reviewer Comment** | **Author Response** |
| --- | --- |
| 1. This manuscript does meet PLOS Digital Health’s publication criteria. This manuscript is clearly written with sound design and provides useful information for researchers planning to use wearables. The conclusion that software updates can impact measurements is important to establish. | Thank you for this comment. |
| 2. The statistical analysis has been performed appropriately and rigorously.  As the software updates arrive in September each year and the activity trends go both up and down with software updates, time of year is somewhat accounted for. Line 169 does explain accounting for this but it is unclear if the 14 day window is fixed or not. Please clarify. | Thank you for bringing this to our attention. The timing of the 14-day period can differ between participants, depending on the date when they upgraded their software version. However, as illustrated in Table 1, approximately half of all software transitions occur within September, which is unsurprising, as software upgrades are released in September.  We have made the following changes to clarify this:  (1) We have added the following sentence to the second paragraph in the “Study design and data collection” section of the methods: “The current study only considered physical activity data collected in the week before and the week after the participant upgraded their hardware or software, the timing of which differed between people.”  (2) We have revised the second paragraph of the “Data processing” paragraph of the Methods to read: “The effect of each software and hardware transition of interest (i.e., from watchOS 5 to 6, 6 to 7, 7 to 8, 8 to 9 and between any two hardware versions) was estimated by comparing daily active calories and exercise minutes in the 7 days before and 7 days after (and including) the participant transitioned between those two software versions. As such, the absolute timing of the transition period differed between participants, depending on the date when they made the software upgrade of interest. In other words, the 14-day period was defined separately for each participant and was centered on the first date that they used the new software or hardware version. This process is illustrated in Figure 1.”  (3) We also created a figure (Figure 1) to outline this process and to help visualize the statistical analysis. |
| It could be useful to analyze the same two weeks in September in the people who did not apply their software updates until November. In a similar way, an analysis of two weeks with the same software at any point could show what a normal deviation week to week might occur. This would provide more confidence when activity significantly changed for males but not females between updates if this trend is not seen when software updates stay the same. | Thank you for this suggestion. We have added a control analysis to address this. In short, the control analysis compares physical activity two weeks before vs. one week before the transition (while the actual analysis compares the week before and the week after the transition).  The control analysis is discussed in the following sections:  Methods:  (a) “Data processing” section:  “To estimate normal week-to-week variability, we also conducted a control analysis for each transition, comparing activity data from 15-8 days before and 7-1 day before that upgrade (“control transition period”) (Figure 1).”  (b) “Descriptive analyses”: “As a control, this was repeated comparing data from 15-8 days before and 7-1 days before each given transition, where no software or hardware upgrades occurred.”  (c) “Regression analyses”: “This analysis was conducted separately for each outcome (daily exercise minutes and daily active calories) and was repeated using data from the pseudo-transition period as a control.”  Results  For each set of results (e.g., the effect of transitioning from watchOS 7 to 8), we follow up with the results of the control analysis for that transition (two weeks before vs. one week before the watchOS 7 to 8 transition). For example, from the first paragraph of the “Software version” section of the results:  “In descriptive analyses, mean daily exercise minutes were, on average, 5.5 minutes higher in the week after vs. the week before upgrading from watchOS 7 to 8 (Supplementary Table 2; Figure 2 Panel A). The average change was larger for female (8.7 minutes) than male participants (3.5 minutes). In mixed effects negative binomial regression models, the watchOS 7 to 8 transition was associated with a 13% increase in daily exercise minutes (unadjusted rate ratio (RR) = 1.13, 95% CI: 1.06, 1.20; adjusted RR = 1.13, 95% CI: 1.06, 1.19). The effect was similar in sex-stratified models (Table 3). **Importantly, there was no significant change in daily exercise minutes in the control analysis comparing data from two weeks before vs. one week before the watchOS 5 to 6 upgrade (Figure 2 Panel B; Table 3).**”  We have also updated Supplementary Table 2 (previously part of Table 1) to include the change in mean daily exercise minutes and mean daily active calories across this pseudo-transition period (2 weeks before vs. one week before) for each transition of interest, and we have updated Figures 2 and 3 (previously Figures 1 and 2) to include the effect of each transition on daily exercise minutes, as well as the associated change in daily exercise minutes in the two weeks before that transition.  We have also added a paragraph to the discussion, highlighting how the null results of the control analysis (for most transitions) strengthens our conclusion that the software transitions may be altering physical activity measurement. |
| 3. No but the authors state that it may be available on request – pending ethical approval. | That is correct. |
| 4. Yes , the manuscript is well written and clear. | Thank you. |
| 5. i. Table 1 displays some general cohort characteristics. However, the mean activity levels and standard deviations for the cohorts analyzed are not shown. Although the changes in activity levels are shown as a percentage and reported in minutes, it would be interesting to understand how active the cohort is to understand how meaningful the % change or 5 minutes reported difference is. | Thank you.  We have created Supplementary Table 2 (previously part of Table 1) to add mean daily exercise minutes and daily active calories (and standard deviation) in the week prior to each transition (e.g., the last week before switching between watchOS 5 and 6). This reflects baseline activity prior to switching to the new software (or hardware) version.  We have also added the following to the final paragraph of the “Sample Characteristics” section: “The cohort was quite active, recording an average of 50 minutes of exercise per day in the week before each upgrade of interest (Table 1). Male participants achieved higher active calories across all transitions, while daily exercise minutes were similar for male and female participants until the week before the watchOS 6 to 7 transition, when sex-differences emerged.” |
| 5. ii. The authors discuss how sex specific algorithms may mean different changes in men and women, but also might want to mention how different mean weights and sizes of males v females might make change more detectable in larger males. | Thank you for this idea.  We have added the following sentence to the third paragraph of the discussion: “Third, the observed changes may be due to updates in how activity algorithms account for weight, which differs on average by sex.” |
| 5. iii. Line 159 – people are excluded if they wear their watch less than 10 hours per day – does this include wearing it when asleep or exclude sleep time? | Our estimate of wear time is the sum of all periods when the user’s Apple Watch was worn (heart rate measurements less than 40 minutes apart), so this would include sleep time if the user wore their watch while sleeping and thus had frequent heart rate measurements during that period. |
| 5. iv. Line 186 and Line 202– Clarifying the use of the terms the primary analysis and the descriptive analysis. Please can you clarify if the descriptive analysis is a secondary analysis that does not require data every day or if descriptive refers to the type of analysis. | We have updated the first sentence of the second and third paragraphs of the “Statistical Analysis” section to clarify what is meant by “descriptive analysis”:  “For our descriptive analysis, we calculated participant-level changes in mean daily exercise minutes and mean daily active calories from the week before to the week after each of the five transitions.”  And  “We also ran a model-based analysis, whereby separate mixed effects negative binomial regression models with a random intercept per participant were fit for each of the five transitions (four software, one hardware), overall and stratified by participant sex.”  Both the descriptive and model-based analyses were run on the primary analytic sample (where complete data was required for both weeks) and as sensitivity analyses, where restrictions on data completeness were loosened. |

**Reviewer #2:**

| **Reviewer Comment** | **Author Response** |
| --- | --- |
| Reviewer #2: This article studies an often neglected issue. However, comparing measurements between software versions does not seem to carry significant input to the research community. The key is still whether these measurements are reasonably accurate proxies to the 'real' data. While confounding of software versions may be an issue, is that a significant issue for the research outcome? Under what scenarios is the versioning impacting the research outcome? | We have revised the following paragraph to make it more clear that changes in measurement due to software or hardware version would also impact the accuracy of those measurements (either increasing or decreasing the degree to which they approximate the “real” data):  “However, hardware and software upgrades may also affect physical activity measurement, posing a significant threat to the validity of research leveraging consumer wearable devices. New hardware versions may contain new and/or improved sensors, while software upgrades could alter activity processing algorithms. This means that the accuracy of physical activity data from wearable devices may vary between people and/or over time depending on software or hardware version. Furthermore, comparisons of physical activity levels over time or between individuals may reflect the combined effects of differences in behaviour and differences in measurement, due to differences in software or hardware versions.”  We also updated the first sentence of the fourth paragraph of the introduction: “While researchers have noted the potential impact of software or hardware version on activity measurement – and thus on research using commercial wearable devices^4^ – no studies have attempted to quantify this effect.” We have referenced the following paper, which clearly identifies hardware and software upgrades as a threat to the validity of research using wearable devices to measure physical activity:  Strain T, Wijndaele K, Pearce M, Brage S. Considerations for the use of consumer-grade wearables and smartphones in population surveillance of physical activity. J Meas Phys Behav. 2022;5(1):8–14.  We also revisit it in the “Research Implications” section of the discussion (“These results have important implications for physical activity research using consumer wearable devices. Most notably, failing to account for software version may introduce bias. Studies comparing physical activity levels between participants or over time, including analyses of longitudinal trends and/or the effect of an intervention, may be confounded by differences in software versions between people and/or over the course of the study period. A large majority of Apple Watch users in our sample installed all major software versions released during their study period. These upgrades are annual, so many software changes should be expected over the course of a multi-year longitudinal study. Based on our estimates, the confounding may be large and either over or underestimate the association of interest. As such, it is important that researchers using data from consumer wearables collect data about software and hardware versions when either varies between participants or over time.”). |

| **Reviewer Comment** | **Author Response** |
| --- | --- |
| Reviewer #3: Overall this is a nice article, well presented, and making an important contribution to the field. | Thank you. |
| Introduction: Well written, provides a good background to the problem. Reflections on existing literature may be a little bit light. | Thank you. |
| Methods: Well written, clearly presented.  Using 40 minutes without an HR measurement to define non-wear time seems to depend on the algorithm Apple is using – and so potentially the software version? If overall results are impacted by non-wear time, could you be detecting an artefact where Apple is changing how it records heart rate but actually its actigraphy measurements remain the same? It is likely not having a big effect, but would be good to know if this has been considered. | Thank you for this interesting idea. We do not think this is an issue. As of 2015, Apple Watch “[attempts to record a heart rate measurement every 10 minutes but won’t record is when you’re in motion or your arm is moving](https://www.theverge.com/2015/5/30/8691513/apple-watch-sporadic-heart-rate-readings-explained)”. This is in addition to continuous monitoring during workouts. We have updated the following sentence to clarify this: “As of 2015, passive measurements were attempted every 10 minutes but were not recorded if the user was in motion^18^, and a recent study found that Apple Watch records a heart rate measurement every 5.62 minutes on average.^19^”. There does not appear to be any documentation indicating that this has changed in subsequent software updates. Based on this information, heart rate measurements should nearly always be less than 40 minutes apart when the Apple Watch is being worn.  Additionally, if a heart rate measurement is recorded after 45 minutes, only those 45 minutes are considered non-wear time. As such, the 40-minute threshold is only problematic if Apple Watch regularly records heart rate measurements at 40+ minute intervals, which does not appear to be the case.  Hopefully this helps. |
| Results: Table 1 is a little bit confusing. The first two sets of rows show outcomes 1 and 2, while the remainder show participant characteristics. I think it would be easier to interpret as two separate tables – with outcomes and participant characteristics separate. | Thank you. We have separated Table 1 into two tables: Table 1 (participant characteristics) and Supplementary Table 2 (outcomes). |
| Discussion: Overall conclusions are valid and research implications are important to the field.  That software changes can affect results differentially depending on sex/gender is an important point. This could be reinforced by further statistical analysis comparing female vs male, I think at the moment it is just based on the averages being different. | Thank you.  We have compared:  (a) the average change in mean daily exercise minutes and mean daily active calories for male and female participants, noting that the average changes are different by sex.  (b) we have also run sex-stratified negative binomial regression models on data from the two week transition period (week and week after upgrade).  In both cases, the results differed by sex.  Interestingly, we have now added a control analysis, where we re-ran the descriptive and regression analyses on data from the two weeks before the transition of interest (comparing two weeks before vs. one week before, where no software or hardware transition had occurred), and the results were similar across sexes, suggesting that there is no significant week-to-week variability for either male or female participants. We believe this strengthens our conclusion that there is at least some evidence that (a) some software upgrades appear to influence activity measurement, and (b) software upgrades may influence male and female user differently. |
